# Evaluation of F18 enterotoxigenic *Escherichia coli* intestinal attachment and early disease onset in nursery pigs

**DOI:** 10.3389/fvets.2025.1686769

**Published:** 2025-12-10

**Authors:** Elizabeth M. Due, Kayla A. Miller, Eric R. Burrough, Emma T. Helm, Nicholas K. Gabler

**Affiliations:** 1 Department of Animal Science, Iowa State University, Ames, IA, United States; 2 Department of Veterinary Diagnostic and Production Animal Medicine, Iowa State University, Ames, IA, United States; 3 School of Animal Sciences, Virginia Polytechnic Institute and State University, Blacksburg, VA, United States

**Keywords:** pig, enterotoxigenic (ETEC), attachment, intestinal integrity, diarrhea

## Abstract

**Introduction:**

Enterotoxigenic *Escherichia coli* (ETEC) is a major cause of post-weaning diarrhea and reduced performance in nursery pigs. While ETEC pathogenesis is well established, the early epithelial and functional responses to F18 ETEC infection remain poorly defined. This study investigated the effects of F18 ETEC on bacterial attachment, intestinal function, and early epithelial cell responses.

**Methods:**

Ten days post-weaning, 24 individually housed pigs (n = 6/treatment) were orally inoculated with 5 ml of F18 ETEC at 10^7^, 10^8^, or 10^9^ colony-forming units (cfu)/ml, or with a negative control (NC). Over a 5-day post-inoculation period, fecal scores, body weight, and growth performance were recorded. Thereafter, pigs were humanely euthanized, ileal contents and fecal F18 and LT gene abundances were quantified, and ileal tissue was assessed *ex vivo* for transepithelial resistance (TER), FITC-dextran permeability (FD4), and active glucose and glutamine transport. Jejunum, ileum, and colon were examined for histomorphology, F18 attachment (*in situ* hybridization), chloride secretion (cystic fibrosis transmembrane conductance regulator [CFTR] protein), and proliferation (Ki67). Ileal gene expression of epithelial proliferation, maturation, and differentiation markers was analyzed.

**Results:**

ETEC-challenged pigs had higher fecal scores than NC (*p* = 0.01), without differences in average daily feed intake or gain:feed (*p* > 0.10). Average daily gain tended to be lower in the 10^8^ ETEC group compared to the NC (*p* = 0.07). In ETEC pigs, F18 and LT gene abundances were elevated (*p* < 0.001) and F18 attachment increased across all intestinal segments (*p* < 0.10), being greatest in the ileum (*p* < 0.001). CFTR protein abundance increased in all regions with ETEC challenge (*p* < 0.05), and Ki67 abundance tended to be lowest in the 10^9^ group (*p* = 0.08). Notch expression tended to increase (*p* = 0.08) and Hes1 tended to decrease (*p* = 0.08) with ETEC challenge, suggesting altered epithelial renewal dynamics. Nutrient transport, TER, and FD4 flux were unaffected (*p* > 0.10).

**Discussion/Conclusion:**

A 5-day F18 ETEC challenge induced ETEC attachment and diarrhea. These findings support a model where F18 ETEC epithelial attachment drives diarrhea through an enterotoxin-mediated, CFTR-dependent secretory mechanism rather than structural epithelial damage.

## Introduction

Enterotoxigenic *Escherichia coli* (ETEC) is a major cause of post-weaning diarrhea (PWD) and edema disease (ED) in nursery pigs globally, posing significant economic burdens due to mortality, morbidity, reduced growth rates, and heightened medication costs (1–3). Enterotoxigenic *Escherichia coli*-induced PWD and ED can occur independently or concurrently, with prevalence varying by pig age and severe manifestations emerging 2 to 3 weeks post-weaning, leading to severe diarrhea or sudden death (1, 4). In pigs, fimbria type and the enterotoxin profile are the two critical virulence factors for ETEC disease (5). Among pigs affected by ETEC-induced PWD, the prevalent adhesive fimbriae types are F4 (K88) and F18 (6). The primary enterotoxins of F18 ETEC include heat-labile toxin (LTI and LTII), heat-stable toxins (STa and STb), enteroaggregative heat-stable toxin 1 (EAST1), and Shiga toxins (Stx1, Stx2, and subtypes) (5, 7). Notably, a Shiga toxin subtype (Stx2e) is associated with porcine ED. (7)

Clinical signs of F18 ETEC-induced PWD and ED typically manifest between 2 to 3 weeks post-weaning, often persisting for up to 10 days (2). Various studies have utilized different inoculation doses of F18 ETEC (8–13) to induce this disease in young pigs. Luise et al. (14) suggested that although pigs may develop diarrhea, a high variability in diarrhea severity and incidence has been observed. This variation is likely due to differences across studies in *α*(1,2)-fucosyltransferase gene (*FUT1*) genetic susceptibility (15), immune competence (16), age (17), and natural exposure to *E. coli* from the sow, the environment, or both (2).

Upon oral ingestion of F18 ETEC in pigs, fimbriae adhere to specific receptors on the apical membrane of enterocytes. Enterotoxins induce the accumulation of cyclic adenosine monophosphate and cyclic guanosine monophosphate, and subsequent changes in the function of the cystic fibrosis transmembrane conductance regulator (CFTR) within the enterocytes, causing disturbances in the electrolyte balance and water flux in the small intestine (1). Thus, the extent of F18 ETEC epithelial adhesion dictates disease severity.

Another commonly reported impact of ETEC disease in pigs is modulation of intestinal morphology, such as decreases in villus height and increases in crypt depth (16, 18), altered barrier integrity through increased transcellular and paracellular permeability (19), or through the disruption of tight junction proteins (20, 21). Previous studies have reported increases in IL6, IL8, and TNF-α in the small intestine of nursery pigs (18, 20, 22), suggesting an inflammatory response that may further exacerbate epithelial damage and contribute to intestinal dysfunction. Intestinal epithelial cell proliferation, maturation, and differentiation, under the control of Notch and Wnt signaling pathways, are needed to maintain and restore intestinal function and integrity (23, 24). Enterotoxins have been shown to increase crypt proliferative stem cells (18) while inhibiting intestinal stem cell differentiation (25). Thus, aspects of epithelial restitution are impacted by F18 ETEC disease.

Early epithelial and functional responses to F18 ETEC attachment remain poorly characterized, despite well-established mechanisms of ETEC pathogenesis. Therefore, the study objectives were to understand the effect of oral F18 ETEC challenge dose on F18 ETEC epithelial attachment and on markers of intestinal function in nursery pigs over a 5-day challenge period. Secondly, this study investigated the effect of early F18 ETEC disease on markers of epithelial cell proliferation, differentiation, and renewal. It was hypothesized that increasing the challenge dose would enhance F18 ETEC attachment to the intestinal epithelium, impairing intestinal function, integrity, and epithelial renewal, and consequently increase diarrhea incidence and reduce nursery pig growth performance.

## Materials and methods

All animal procedures were approved by the Iowa State University Institutional Animal Care and Use Committee (IACUC protocol #22–123) and adhered to the ethical and humane use of animals for research.

### Experimental design, housing, and inoculations

Twenty-four mixed-sex freshly weaned pigs (5.7 ± 0.44 kg body weight, 19–21 days of age; Camborough (1050) × PIC 337 (Hendersonville, TN) were randomly selected and placed in individual pens in a single room. For all pigs, the *FUT1* genotype was determined from ear notches submitted to the Iowa State University Veterinary Diagnostic Laboratory (ISU VDL) in Ames, IA, for PCR genotyping as described by Frydendahl et al. (26), and *FUT1* genotypes of AG and GG were considered susceptible.

Each pen (122 × 42 × 61 cm) was equipped with its own nipple waterer and feeder. The room was maintained at a 12/12-h light/dark cycle and was temperature controlled within the thermoneutral zone of the pigs for the duration of the experiment. All pigs were fed a non-medicated, corn-soybean meal-based mash diet, devoid of antimicrobial ingredients such as antibiotics and zinc oxide, in two dietary phases that met or exceeded energy and nutrient requirements for this size of pig (55). The phase 1 diet was formulated to a standard ileal digestible (SID) lysine of 1.45% and contained 3,400 kcal/kg metabolizable energy (ME) and was fed from placement, or day post-inoculation (dpi) -10 to 0 dpi. The phase 2 diet was formulated to contain 1.38% SID lysine, and 3,400 kcal/kg ME and was fed from 0 to 5 dpi. Pigs were provided *ad libitum* access to feed and water for the duration of the experiment.

Upon arrival, pigs were randomly allotted to 4 treatment groups (n = 6 pigs/treatment): (1) non-inoculated (NC), or F18 ETEC inoculated with either, (2) 10^7^ colony-forming units (cfu) per mlml, (3) 10^8^ cfu/ml, or 4) 10^9^ cfu/ml. On −1 dpi, fecal samples were taken and submitted to the ISU VDL for confirmation of negative hemolytic *E. coli* status via routine culture. Following the 10 days of acclimation (0 dpi), the 10^7^, 10^8^, and 10^9^ cfu treatment groups were orally inoculated with a 5-ml F18 ETEC (EAST1, LT, STb, Stx2, STx2e positive) challenge. On 2 dpi, pigs were reinoculated with 5 ml of inoculum administered into their individual feeders and 1 ml onto waterspouts. The F18 ETEC inoculum was grown and diluted in Luria broth cultures, and cfu treatment dilutions were confirmed through optical density and via viable cfu plate counts (27).

Daily feed consumption was measured for the duration of the experiment by weighing feeders and calculating feed disappearance. Fecal scores were recorded daily by one individual during both the acclimation and challenge periods using a scale of 0 = solid, 1 = semi-solid, 2 = soft, 3 = liquid. Body weights were recorded at placement (−10 dpi), 0 dpi, and 5 dpi to calculate average daily gain (ADG), average daily feed intake (ADFI), and gain:feed.

### Sample collection

Fecal samples were collected from all individual pigs at −1, 2, and 5 dpi. At 5 dpi, after all challenged pigs had at least 3 consecutive days of scouring, all pigs were euthanized via captive bolt followed by exsanguination for tissue and luminal contents collection. Ileal contents and fecal samples were collected for assessment of *F18* and *LT* gene abundance via PCR. Ileal sections were collected 30–40 cm proximal from the ileal-cecal junction. Luminal digesta pH measurements were promptly taken at necropsy using pH indicator strips (ThermoFisher Scientific, Waltham, MA, USA) ranging from 5.0 to 9.0 for jejunum, ileum, and colon, and from 0.0 to 14.0 for stomach digesta.

Distal jejunum, distal ileum, and apex colon tissues were excised and placed into 10% neutral buffered formalin for fixation. Fixed samples were moved to 70% ethanol after approximately 24 h of fixation and were later submitted to the ISU VDL to be paraffin-embedded, sectioned, and mounted onto slides for routine histological staining and evaluation as described below. Fresh ileum sections were also flushed with Krebs–Henseleit buffer (25 mM NaHCO_3_, 120 mM NaCl, 1 mM MgSO_4_, 6.3 mM KCl, 2 mM CaCl_2_, and 0.32 mM NaH_2_PO_4_) and snap-frozen in liquid nitrogen until further analysis.

### *Ex vivo* assessment of ileal barrier function and integrity

A section of fresh ileum was excised immediately post-euthanasia and placed into continuously aerated ice-cold Krebs–Henseleit buffer, transported to the lab, and mounted in modified Ussing chambers (Physiological Instruments, San Diego, CA) as previously described by (28). The live ileal tissues were mounted in modified Ussing chambers within 1 h of euthanasia to determine intestinal barrier integrity and ileal active transport. The modified Ussing chamber apparatus was fully assembled prior to tissues arriving, with current and voltage electrodes filled with 3% noble agar and submerged in 3 M KCl, chambers filled with KB, and the system heated and leaks eliminated (28, 29). Voltage differences between chambers were offset, and fluid resistance compensation was used to account for non-tissue-related resistance to ensure all chambers had a baseline measurement between 60 and 65 μA. Ileum segments were pinned onto inserts that allowed for an exposed surface area of 0.71 cm^2^ with serosal and mucosal membranes facing opposite chambers. Serosal and mucosal sides were bathed in 4 ml Krebs–Henseleit buffer, and tissue segments were provided with a constant mixture of 95% O_2_–5% CO_2_. Intestinal segments were voltage clamped at 0 mV by an external current after correction for solution resistance and stabilized for a 30-min period (30). After stabilization, transepithelial resistance (TER) was measured as ohms (*Ω*) per cm^2^, and mucosal to serosal flux of 4.4 kDa FITC-Dextran (FD4; Sigma Chemical, St. Louis, MO) measurements were collected. A fluorescent plate reader (Cytation 5 Hybrid Multi-Mode Reader, BioTek Instruments, Inc., Winooski, VT) was used to determine changes in relative fluorescence of FD4 in serosal samples from 0 to 60 min after FD4 addition at 485 and 520 nm excitation and emission wavelengths, respectively, and a permeability coefficient was then calculated (28, 29). Active mucosal to serosal transport of glucose and glutamine was determined in the ileum samples as previously described (30).

### Intestinal morphology, immunohistochemistry, immunofluorescence, and *in situ* hybridization

Fixed sections of distal jejunum, ileum, and apex colon tissue were paraffin-embedded, sectioned, mounted onto glass slides, and stained with hematoxylin and eosin at the ISU VDL for evaluation of intestinal morphology. In these stained sections, 15–20 well-orientated villus (jejunum and ileum) and crypt (jejunum, ileum, and colon) pairs were measured for height and depth, respectively, in μm using OLYMPUS CellSens Dimension 1.16 software (Olympus Scientific, Waltham, MA). Measurements of villus height (μm) and crypt depth (μm) were used to determine villus-to-crypt ratios (VCR), previously described by Helm et al. (31).

Immunohistochemistry (IHC) and immunofluorescence assay (IFA) were performed to evaluate epithelial proliferation and chloride secretion, respectively, in the jejunum, ileum, and colon of NC, 10^7^, 10^8^, and 10^9^ pigs at 5 dpi. For IHC, an anti-Ki67 antibody (Dako, Glostrup, Denmark) was used to detect proliferating stem cells within intestinal crypts, following procedures described by Curry et al. (32). For the IFA, a rabbit polyclonal anti-CFTR antibody (PA5-121193; Thermo Fisher Scientific, Waltham, MA, USA) was applied at a 1:200 dilution, followed by a goat anti-rabbit IgG (H + L) secondary antibody conjugated to Alexa Fluor 488 (A32732; Thermo Fisher Scientific, Waltham, MA, USA) at a 1:500 dilution. Nuclei were counterstained with DAPI (1:1000). IHC and IFA slides were imaged at 20X (Olympus Scientific, Waltham, MA), and images were analyzed in the HALO software (v2.0.1145.19, Indica Labs, Chico, CA) for analysis as previously described (32). Positive Ki67 and CFTR staining were calculated and reported as a percentage of positive area to total tissue area.

Chromogenic RNA *in situ* hybridization (ISH) for *F18* transcripts was performed using a RNAScope® 2.5 probe (GeneBank Accession M61713.1, catalog #049871-C1, Advanced Cell Diagnostics, Hayward, CA, USA), according to the manufacturer’s instructions at the ISU VDL. Briefly, blocks of formalin-fixed paraffin-embedded ileum samples were sectioned at 4 μm, mounted onto positively charged slides, and dried overnight at room temperature. Slides were then de-paraffinized by passage through xylene (2 × 5 min) and dehydrated by immersing in 100% alcohol (2 × 1 min). The slides were air-dried and quenched with RNAscope hydrogen peroxide at room temperature for 10 min and rinsed with distilled water. After immersing in the prepared RNAscope 1X Target Retrieval Reagent for 15 min at 100 °C, tissue slides were rinsed in distilled water, immersed in 100% alcohol for 3 min and then air-dried at room temperature. Slides were then incubated with RNAscope Protease Plus for 30 min at 40 °C in the HybEZTM Oven and rinsed in distilled water 3 times. The slides were incubated with the preheated buffer containing the specific RNA probe and hybridized for 2 h at 40 °C in the HybEZTM Oven. Following amplification of six rounds in hybridization buffer, slides were washed, incubated with red chromogenic detection solution for 10 min at room temperature, counterstained with hematoxylin, and finally cover slipped with mounting media. Appropriate controls were present in every run, including the positive control probe-Ss PPIB (ACD RNAscope) and negative control probe-dapB (ACD RNAscope).

Slides labeled by RNA ISH were imaged using an Olympus DP74 camera mounted on an Olympus BX41 microscope with UPlanFLN objectives and operated with CellSens Dimension software (version 1.17, Olympus Corporation), as previously described by Lin et al. (33) and Helm et al. (34). Representative 400X images of ileal mucosa were captured for each slide and analyzed quantitatively using the Area Quantification module v1.0 within the HALO image analysis platform (v2.0.1145.19, Indica Labs, Chico, CA). Signal was quantified as the percent F18-positive stain area within the region of interest, normalized to the total epithelial area of each epithelial region of interest.

### Gene abundance

Fresh fecal and ileal contents were collected from all pigs at 0 and 5 dpi. These samples were immediately placed on ice and then stored at −80 °C until DNA extraction. Total DNA was isolated from 0.25 g of the sample using a QIAamp® PowerFecal® Pro DNA Kit (QIAGEN, Germantown, MD, USA) following the manufacturer’s instructions. DNA concentrations and purity were verified using a spectrophotometer (Cytation 5 Hybrid Multi-Mode Reader, BioTek Instruments, Inc., Winooski, VT). All samples had a 260/280 ratio of 1.8–2.0. Fecal and ileal DNA samples were used for quantitative PCR (qPCR) using TaqMan Fast Advanced Master Mix (ThermoFisher Scientific, Waltham, MA, USA) with 20X gene-specific primers for *F18* and *LT*. The sequences for the *F18* and *LT* primer sets were as follows: *F18* forward primer (5′-3′): GCGTCGAATAGCACTGTAAGTTTC, *F18* reverse primer: ACCACCTTTCAGTTGAGCAGTAAAT, *LT* forward primer (5′-3′): GCAGGCAAAAGAGAAATGGTTATCA, and *LT* reverse primer: CCGGGACTTCGACCTGAA (ThermoFisher Scientific, Waltham, MA, USA). Each 20 μl sample reaction contained 10 μl of TaqMan Fast Advanced Master Mix, 1 μl of *F18* primer, 1 μl of *LT* primer, 1 μl of template DNA (50 ng/μl), and 7 μl of nuclease-free water. Fluorescence was quantified using a QuantStudio™ 3 Real-Time PCR System (ThermoFisher Scientific, Waltham, MA, USA) with the following conditions: 20-s hold for polymerase activation at 95 °C followed by 40 RT-PCR cycles (denature at 95 °C for 1 s, anneal/extend at 60 °C for 20 s). QuantStudio™ Design & Analysis Software (ThermoFisher Scientific, Waltham, MA, USA) was used to analyze amplification plot, and cycle threshold (Ct) values for each reaction were determined. A reverse Ct value was then calculated as the difference between the Ct value of the gene of interest and the maximum Ct of 40 amplification cycles for the assay.

Total RNA was extracted from frozen ileal tissue according to the Trizol protocol (Invitrogen, Grand Island, NY). Quantity and purity of extracted mRNA were determined spectrophotometrically using a Cytation 5 Hybrid Multi-Mode Reader (BioTek Instruments Inc., Winooski, VT, USA). All samples had a 260/280 ratio of at least 1.8. The mRNA was transcribed to cDNA with a commercially available kit (High-Capacity RNA-to-cDNA™ Kit; ThermoFisher Scientific, Waltham, MA, USA), and the cDNA was used for real-time qPCR (RT-qPCR) on a QuantStudio™ 3 Real-Time PCR System (ThermoFisher Scientific, Waltham, MA, USA). Each 20 μl sample reaction contained 10 μl of PowerUp™ SYBR™ Green Master Mix (ThermoFisher Scientific, Waltham, MA, USA), 1 μl forward primer, 1 μl reverse primer, 2 μl of template cDNA (250 ng/μl), and 6 μl of nuclease-free water. Abundance values were normalized to a reference gene (GAPDH) and NC pigs according to the 2^−ΔΔCt^ method. Gene symbols and primer sequences are listed in Additional File 1.

### Statistical analysis

Data were analyzed using the MIXED procedure of SAS v. 9.4 (SAS Institute, Cary, NC). Pre-challenge (−10–0 dpi) and post-challenge (0–5 dpi) periods were evaluated for main effects of treatment (NC, 10^7^, 10^8^, 10^9^) and challenge (NC and ETEC Challenge). RNAscope ISH *E. coli F18*, IHC (Ki67), IFA (CFTR), and RT-qPCR data were log-transformed for normality. The main effect of tissue (jejunum, ileum, and colon) was also assessed for RNAscope ISH *E. coli F18*, IHC (Ki67), and IFA (CFTR) data. Least square means were obtained using the LSMEANS statement with the Tukey adjustment, and results are presented as least square means with a pooled standard error. Ordinal fecal consistency scores were analyzed for treatment-by-day and challenge-by-day interactions using PROC GENMOD in SAS v. 9.4 (SAS Institute, Cary, NC) with a multinomial distribution and cumulative logit link, specifying pig as the repeated subject and an autoregressive [AR(1)] correlation structure. *p*-values were calculated using chi-squared tests for type 3 generalized estimating equations. For all analyses, differences were considered significant at *p* ≤ 0.05, and a tendency when *p* ≤ 0.10.

## Results

### Pig health, fecal scores, and F18 ETEC burden

**Figure 1:**
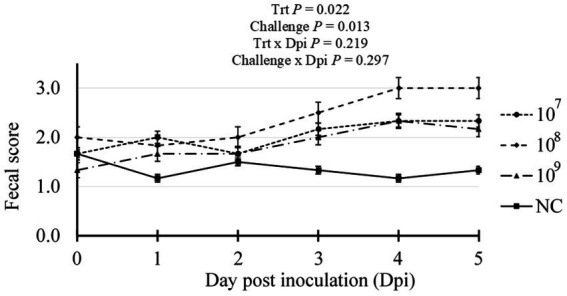
Daily fecal scores of pigs challenged with either 0 (Negative control, NC), 10^7^, 10^8^, or 10^9^ cfu F18 enterotoxigenic *Escherichia coli* (ETEC). *N* = 6 pigs/treatment. Challenge *p*-value represents NC vs. ETEC challenge (10^7^, 10^8^, 10^9^ cfu). Fecal scoring: 0 = solid, 1 = semi-solid, 2 = soft, 3 = liquid stool.

Initial assessment of fecal samples prior to inoculation confirmed the absence of hemolytic *E. coli* and the presence of porcine rotavirus as determined via routine enteric diagnostics by the ISU VDL (data not shown). Over the study duration, no antimicrobial interventions were administered, and one mortality was recorded within treatment 10^8^ between 4 and 5 dpi. Fecal scouring was assessed daily post-inoculation (Figure 1). Compared to the NC treatment, all pigs challenged with ETEC had significantly increased scour scores, indicative of diarrhea, with the 10^8^ treatment exhibiting a higher average daily score incidence (Trt *p* = 0.030). Compared to the NC treatment group, all F18 ETEC-challenged pigs had higher fecal scores, indicative of loose or soft fecal consistency (Challenge *p* = 0.013).

**Figure 2:**
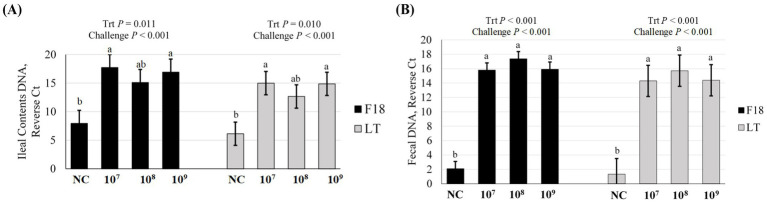
Gene abundance of *F18* and *LT* in **(A)** ileal contents and **(B)** fecal contents of pigs at day post inoculation 5, following challenge with F18 enterotoxigenic *Escherichia coli* (ETEC) with either 0 (Negative control, NC), 10^7^, 10^8^, or 10^9^ cfu. *N* = 6 pigs/treatment. Challenge *p*-value represents NC vs. ETEC challenge (10^7^, 10^8^, 10^9^ cfu). Means reported as reverse Ct, or the difference in Ct value for the gene of interest and the maximum Ct value for the assay of 40 amplification cycles. Different letters a, b represent *p* < 0.05.

To further confirm the F18 ETEC challenge, *F18* and *LT* gene abundances were assessed in ileal and feces contents at 5 dpi by PCR (Figure 2). The ileal contents *F18* abundance was higher, as denoted by an increased reverse Ct, in all ETEC challenge groups compared to NC (*p* < 0.001), with the 10^7^ and 10^9^ treatments exhibiting the highest *F18* abundance (*p* = 0.011, Figure 2A). Similarly, *LT* toxin abundance in ileal contents was elevated in ETEC challenge groups compared to NC (*p* < 0.001) and again was highest in the 10^7^ and 10^9^ treatments (*p* = 0.010). In feces at 5 dpi (Figure 2B), both the *F18* and *LT* toxin abundance were elevated in the challenge groups compared to the NC (*p* < 0.001), but were similar among the 10^7^, 10^8^, and 10^9^ ETEC treatments (*p* < 0.001).

**Figure 3:**
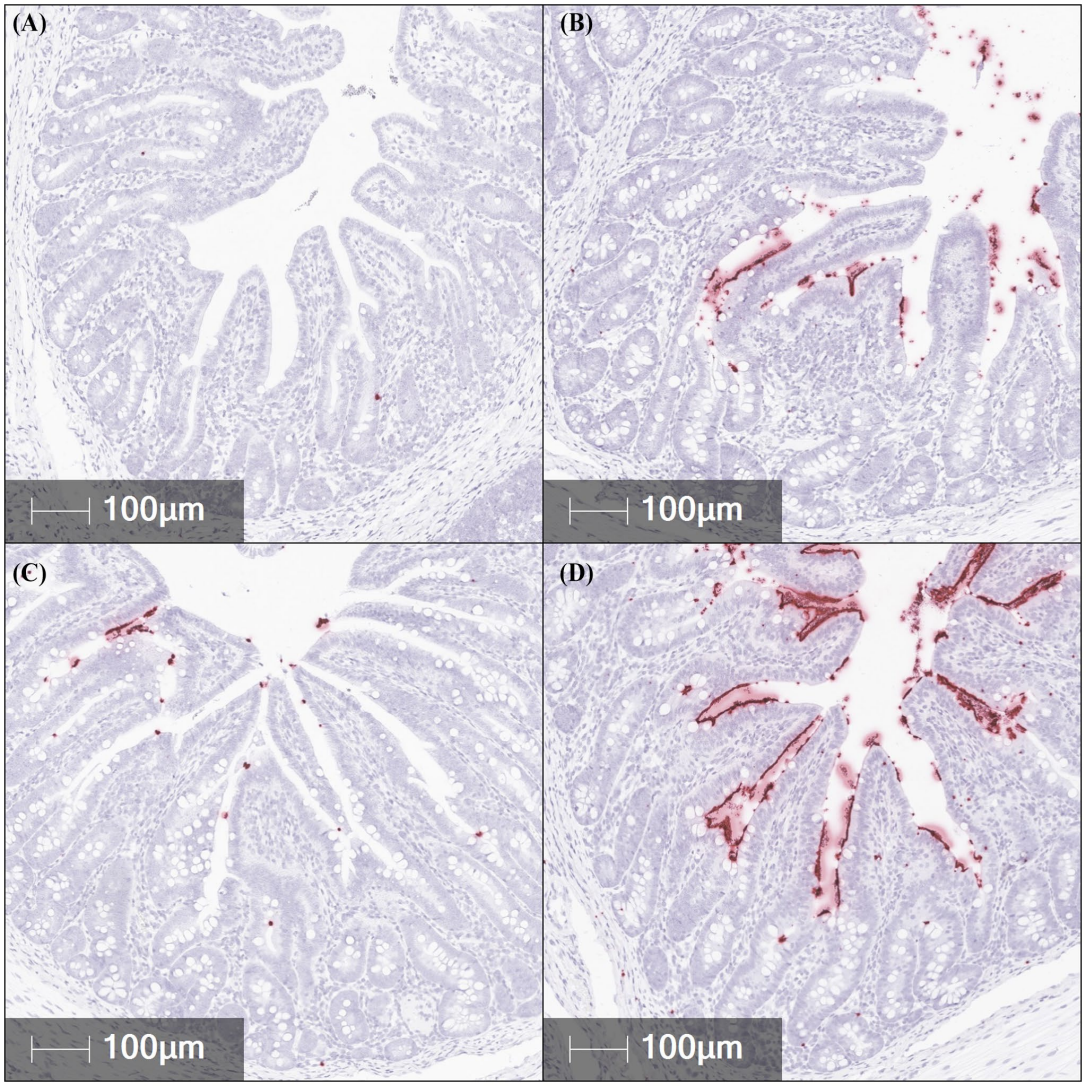
Representative *in situ* hybridization images of *F18* transcript abundance (red staining) in fixed ileal tissues from pigs challenged with F18 enterotoxigenic *Escherichia coli* at 5 dpi. **(A–D)** represent: Negative control (0 cfu, **A**—top left), 10^7^ cfu (**B**—top right), 10^8^ cfu (**C**—bottom left), and 10^9^ cfu (**D**—bottom right). N = 6 pigs/treatment.

In order for the disease to be fully expressed in pigs, F18 ETEC needs to be attached to the intestinal epithelium. *In situ* hybridization (ISH) was used to quantify the abundance of jejunal, ileal, and colonic F18 ETEC attachment at 5 dpi by assessing *F18* transcript localization to the epithelium (Table 1; Figure 3). Compared to NC pigs, ETEC-challenged pigs had higher jejunal *F18* transcript abundance (*p* = 0.040) and tended to have higher abundance in the colon (*p* = 0.094). Ileal *F18* transcript abundance was greater in the 10^7^, 10^8^, and 10^9^ groups than in NC (*p* < 0.001). Across intestinal tissues, ileal sections had the greatest *F18* transcript localization, followed by the jejunum and colon tissue (Tissue *p* < 0.001). Overall, ETEC challenge resulted in approximately 85-fold more F18-positive staining in the ileum relative to NC pigs (*p* < 0.0001).

**Table 1:** Jejunum, ileum, and colon tissue section F18 transcript abundance, CFTR protein abundance, and Ki67 protein abundance in the pigs challenged with either 0 (NC), 10^7^, 10^8^, or 10^9^ cfu of F18 enterotoxigenic *Escherichia coli* (ETEC) after 5 days.

Item	ETEC Challenge^1^, cfu				SEM	*p*-values		
	NC	10^7^	10^8^	10^9^		Trt	Challenge^*^	Tissue^‡^
F18^†^, % positive area								
Jejunum	0.00	0.04	0.17	0.37	0.203	0.241	0.040	<0.001
Ileum	0.01ᵇ	0.89ᵃ	0.63ᵃ	1.28ᵃ	0.452	0.001	<0.001	
Colon	0.01	0.05	0.03	0.03	0.019	0.207	0.094	
CFTR^†^, % positive area								
Jejunum	0.07ᵇ	0.26ᵃᵇ	0.22ᵃᵇ	0.75ᵃ	0.240	0.030	0.003	0.223
Ileum	0.14ᵇ	0.96ᵃ	0.74ᵃᵇ	1.17ᵃ	0.413	0.024	0.003	
Colon	0.19ʸ	0.27ˣʸ	0.44ˣ	0.43ˣ	0.124	0.089	0.043	
Ki67, % positive area								
Jejunum	16.7	17.5	17.8	16.0	1.11	0.596	0.774	<0.001
Ileum	19.0ˣ	17.4ˣʸ	19.3ˣ	14.2ʸ	1.58	0.084	0.240	
Colon	59.3ˣʸ	60.6ˣʸ	62.3ˣ	49.6ʸ	3.92	0.056	0.688	

^a,b^Means with differing superscripts differ significantly at *p* < 0.05. ^x,y^Means with differing superscripts tend to differ at *p* < 0.10.

1 *n* = 6 pigs/treatment.

* NC vs. ETEC challenge (10^7^, 10^8^, 10^9^).

‡ Main effect of intestinal tissue region (Jejunum, ileum, colon).

† *p*-value based on log-transformed value.

### Growth performance

Pig body weights pre- (−10 and 0 dpi) and post-challenge (5 dpi) are reported in Table 2. At −10, 0, and 5 dpi, body weight did not differ by treatment or challenge (*p* > 0.10). No differences in ADG, ADFI, or G:F among treatments or challenge were reported prior to the F18 ETEC challenge (−10 to 0 dpi; *p* > 0.10). Further, over the 5-day challenge period, no differences in ADFI and G:F were observed for treatment or challenge (*p* > 0.10). Compared to the NC, irrespective of F18 ETEC challenge dose, ADG did not differ (challenge *p* = 0.348). However, post-challenge ADG tended to decrease in the 10^8^ treatment group compared to the NC, 10^7^, and 10^9^ treatments (*p* = 0.071).

**Table 2:** Pig body weight and performance parameters pre- and post-F18 enterotoxigenic *Escherichia coli* (ETEC) inoculation challenge.

Item	ETEC Challenge^1^, cfu				SEM	*p*-values	
	NC	10^7^	10^8^	10^9^		Trt	Challenge^*^
Body weight, kg							
Day post-inoculation −10	5.6	5.8	5.7	5.5	0.19	0.624	0.767
Day post-inoculation 0	6.3	6.8	6.6	6.7	0.45	0.859	0.422
Day post-inoculation 5	7.8	8.2	7.1	8.3	0.58	0.507	0.917
Day post-inoculation 0 to 5							
ADG, kg/d	0.26^x^	0.24^x^	0.10^y^	0.26^x^	0.047	0.071	0.349
ADFI, kg/d	0.34	0.36	0.30	0.41	0.045	0.406	0.700
Gain:feed	0.79	0.44	0.32	0.65	0.164	0.205	0.105

^x,y^Means with differing superscripts tend to differ at *p* < 0.10.

1 *n* = 6 pigs/treatment.

* NC vs. ETEC challenge (10^7^, 10^8^, 10^9^).

### Intestinal integrity, nutrient transport, and morphology

Gastrointestinal pH and intestinal morphology were assessed at 5 dpi and reported in Table 3. Stomach pH was elevated in the 10^7^ and 10^9^ treatments compared to the NC and 10^8^ treatments (*p* = 0.047). Ileal pH tended to be higher in F18 ETEC-challenged pigs relative to NC pigs (*p* = 0.055). Specifically, the 10^9^ treatment tended to have a higher ileal pH than NC, with 10^7^ and 10^8^ treatments as intermediate (*p* = 0.077). Jejunal and colonic contents pH were not different among treatment or challenge (*p* > 0.10). Villus height in the jejunum was reduced in treatments 10^8^ and 10^9^ compared to NC (*p* = 0.006) and was also lower in ETEC-challenged pigs overall relative to NC (*p* = 0.013). Jejunum crypt depth did not differ (*p* > 0.10), but VCR in the jejunum was decreased in the 10^9^ treatment group compared to NC (*p* = 0.034) and was lower in challenged pigs relative to NC (*p* = 0.012). Ileal morphological assessment of villus length, crypt depth, VCR, and colonic crypt depth was not different by treatment or challenge (*p* > 0.10).

**Table 3:** Luminal pH and intestinal morphology in the stomach, jejunum, ileum, and colon of pigs challenged with either 0 (NC), 10^7^, 10^8^, or 10^9^ cfu of F18 enterotoxigenic *Escherichia coli* (ETEC) after 5 days.

Item	ETEC Challenge^1^, cfu				SEM	*p*-values	
	NC	10^7^	10^8^	10^9^		Trt	Challenge^*^
Stomach pH	3.7^y^	4.0^x^	3.6^y^	4.0^x^	0.13	0.047	0.165
Jejunum							
pH	6.0	6.3	6.1	6.0	0.18	0.676	0.537
Villus height, μm	452^a^	428^ab^	380^b^	363^b^	18.8	0.006	0.013
Crypt depth, μm	309	333	320	326	14.6	0.657	0.262
Villus:crypt ratio	1.5^a^	1.3^ab^	1.2^ab^	1.1^b^	0.09	0.034	0.012
Ileum							
pH	7.3^y^	7.5^xy^	7.6^xy^	7.8^x^	0.14	0.077	0.055
Villus height, μm	352	357	305	384	31.1	0.342	0.991
Crypt depth, μm	248	236	219	262	17.9	0.362	0.691
Villus:crypt ratio	1.4	1.5	1.4	1.5	0.13	0.906	0.693
Colon							
pH	6.7	6.9	6.7	6.5	0.28	0.717	0.894
Crypt depth, μm	493	471	500	462	33.5	0.815	0.637

^a,b^Means with differing superscripts differ significantly at *p* < 0.05. ^x,y^Means with differing superscripts tend to differ at *p* < 0.10.

1 *n* = 6 pigs/treatment.

* NC vs. ETEC challenge (10^7^, 10^8^, 10^9^).

**Figure 4:**
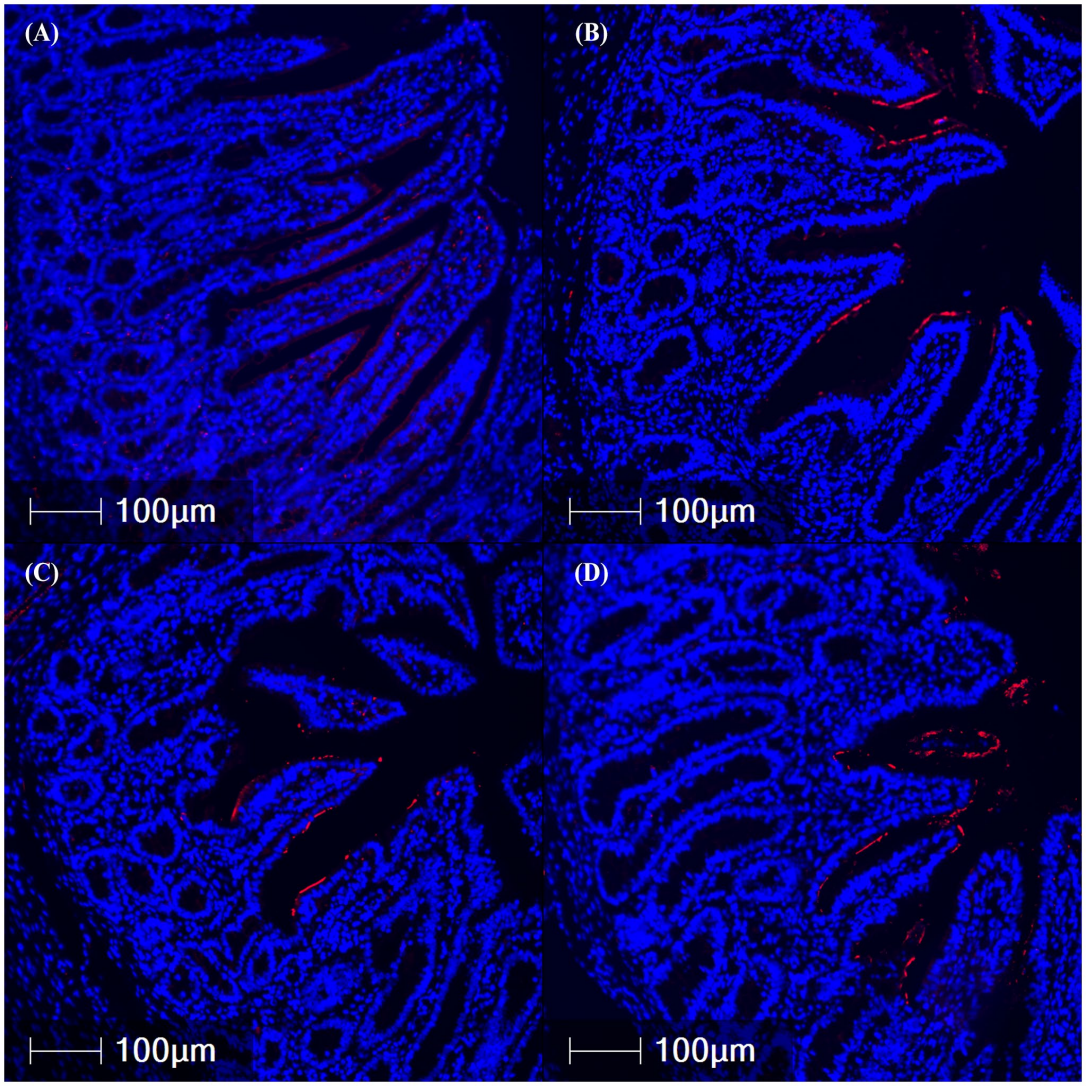
Representative images of ileal cystic fibrosis transmembrane conductance regulator protein abundance (red fluorescence) in fixed tissues from pigs challenged with F18 enterotoxigenic *Escherichia coli* at 5 dpi. **(A–D)** represent: Negative control (0 cfu, **A**—top left), 10^7^ cfu (**B**—top right), 10^8^ cfu (**C**—bottom left), and 10^9^ cfu (**D**—bottom right). N = 6 pigs/treatment.

Total CFTR protein was assessed in the jejunum, ileum, and colon via IFA (Table 1; Figure 4). CFTR protein abundance was elevated in the jejunum of ETEC-challenged pigs compared to NC pigs (*p* = 0.003) and was highest in the 10^9^ treatment compared to NC (*p* = 0.030). Similarly, total CFTR protein in the ileum was elevated in ETEC-challenged pigs compared to NC (*p* = 0.003), consistent with the hypothesized increase in diarrheal incidence. Specifically, 10^7^ and 10^9^ treatments had greater total ileal CFTR protein than NC (*p* = 0.024). Total CFTR protein in the colon was higher in ETEC-challenged pigs compared to NC pigs (*p* = 0.043) and tended to be increased in 10^8^ and 10^9^ treatments relative to NC (*p* = 0.088). CFTR protein abundance did not differ between tissue regions (*p* > 0.10).

**Figure 5:**
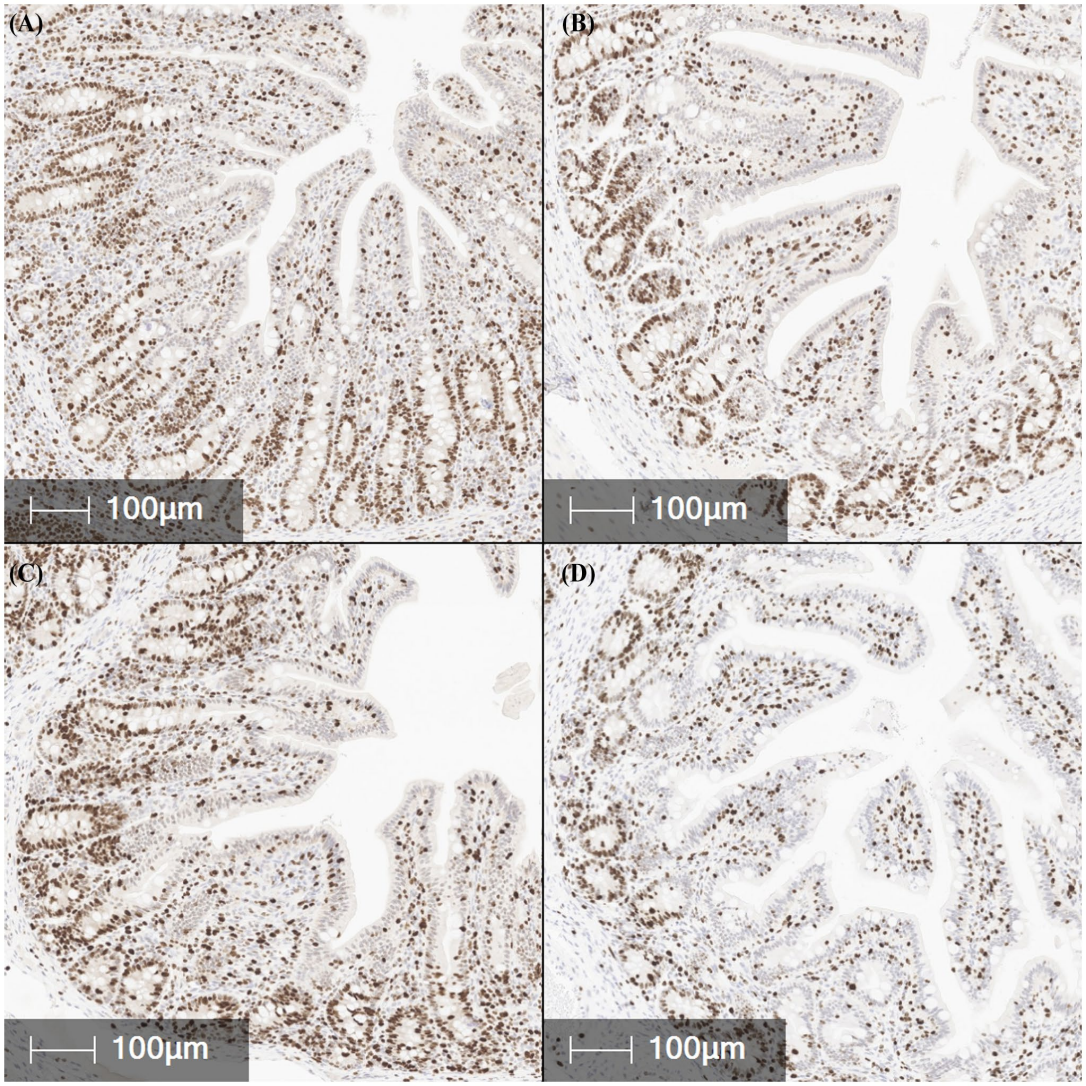
Representative images of Ki67 positive expression (brown staining), a marker of cell proliferation, in fixed ileal tissues from pigs challenged with F18 enterotoxigenic *Escherichia coli* at 5 dpi. **(A–D)** represent: Negative control (0 cfu, **A**—top left), 10^7^ cfu (**B**—top right), 10^8^ cfu (**C**—bottom left), and 10^9^ cfu (**D**—bottom right). N = 6 pigs/treatment.

The abundance of the cell proliferation marker Ki67 was measured via IHC in the jejunum, ileum, and colon tissues (Table 1; Figure 5). The percentage of Ki67-positive stained area in the jejunum did not differ between treatments or challenge groups (*p* > 0.10). In contrast, treatment 10^9^ showed a trend toward reduced Ki67-positive area in the ileum compared to NC and treatment 10^8^, suggesting decreased cell proliferation and maturation (*p* = 0.084). Colonic Ki67 tended to be lowest in the 10^9^ and highest in the 10^8^ treatments, with the NC and 10^7^ treatments being intermediate (*p* = 0.056). Ki67-positive area was highest in the colon compared to the jejunum and ileum (*p* < 0.001).

As ileum tissue was the primary site for F18 attachment, it was hypothesized that *ex vivo* ileal integrity and function, assessed via modified Ussing chambers on dpi 5 (Table 4), would be reduced as ETEC challenge dose increased. However, ileal TER and mucosal to serosal FD4 macromolecule permeability did not differ among treatment or challenge groups (*p* > 0.10). In addition, active glucose and glutamine transport were not different among treatments (*p* > 0.10). These results indicate that, contrary to the hypothesis, F18 ETEC challenge did not compromise epithelial integrity or nutrient transport capacity at dpi 5.

**Table 4:** *Ex vivo* ileum barrier integrity and active transport function in pigs after a 5-day 0 (negative control, NC), 10^7^, 10^8^, or 10^9^ cfu F18 enterotoxigenic *Escherichia coli* (ETEC) challenge.

Item	ETEC Challenge^1^, cfu				SEM	*p*-values	
	NC	10^7^	10^8^	10^9^		Trt	Challenge^*^
Transepithelial resistance, Ω x cm^2^	54	65	63	53	7.8	0.565	0.466
FD4 permeability^‡^	253	325	300	291	36.3	0.550	0.204
Glucose transport, ΔIsc (μA/cm^2^)	28	23	20	35	7.2	0.489	0.794
Glutamine transport, ΔIsc (μA/cm^2^)	23	14	20	13	5.9	0.518	0.226

1 *n* = 6 pigs/treatment.

* NC vs. ETEC challenge (10^7^, 10^8^, 10^9^).

‡ 4 kDa Fluorescein isothiocyanate-dextran (FD4) apparent permeability coefficient flux.

### Ileal mRNA abundance of nutrient and ion transport and intestinal function markers

Ileal tissue nutrient and ion transport, function, proliferation, and differentiation marker gene abundance were quantified using qPCR (Table 5). As ETEC induces secretory diarrhea, we hypothesized that water and ion transporter genes would be upregulated compared to the NC as ETEC challenge increased. Among markers of water and ion transport, *AQP3* expression did not differ between treatments or challenge groups (*p* > 0.10). *Cldn2* expression tended to be decreased in treatment 10^8^ compared to 10^7^ (*p* = 0.052), while *CFTR* expression tended to be increased in treatment 10^9^ compared to 10^8^ (*p* = 0.095). *SGLT1* expression was significantly increased in ETEC-challenged pigs compared to NC pigs (*p* = 0.043). It was further hypothesized that epithelial renewal would be impaired by ETEC challenge. Gene markers of stem cell proliferation, including *WNT4* and *β-catenin*, did not differ between treatments or challenge groups (*p* > 0.10). Among markers of cell differentiation, *Notch* expression tended to be upregulated in ETEC-challenged pigs compared to NC (*p* = 0.081), while *Hes1* expression tended to be reduced (*p* = 0.083), primarily driven by a decrease in treatment 10^9^ relative to NC (*p* = 0.045). No differences were observed in *ATOH1* or *Muc2* expression across treatments or challenge groups (*p* > 0.10). Additionally, *IL8* expression did not differ among any treatment or challenge groups (*p* > 0.10).

**Table 5:** Ileal mRNA abundance in pigs at day post-inoculation 5 following a F18 enterotoxigenic *Escherichia coli* (ETEC) challenge with either 0 (negative control, NC), 10^7^, 10^8^, or 10^9^ cfu.

Gene	ETEC Challenge^1^, cfu				SEM	*p*-values	
	NC	10^7^	10^8^	10^9^		Trt	Challenge^*^
CFTR^†^	1.00^xy^	0.87^xy^	0.69^y^	1.39^x^	0.198	0.095	0.705
AQP3^†^	1.00	1.63	1.43	3.46	0.501	0.266	0.137
Cldn2^†^	1.00^xy^	1.23^x^	0.38^y^	0.58^xy^	0.298	0.052	0.284
SGLT1	1.00	2.33	1.83	2.11	0.465	0.212	0.043
WNT4^†^	1.00	1.35	2.45	1.49	0.466	0.198	0.129
β-catenin^†^	1.00	1.67	0.97	0.95	0.278	0.234	0.567
Notch^†^	1.00	2.02	2.14	1.20	0.472	0.134	0.081
Hes1	1.00^a^	0.82^ab^	0.93^ab^	0.58^b^	0.112	0.045	0.083
ATOH1	1.00	1.06	1.38	1.41	0.153	0.119	0.115
MUC2^†^	1.00	0.82	0.99	1.20	0.327	0.962	0.790
IL8	1.00	0.94	0.73	0.99	0.199	0.843	0.784

^a,b^Means with differing superscripts differ significantly at *p* < 0.05. ^x,y^Means with differing superscripts tend to differ at *p* < 0.10.

1 *n* = 6 pigs/treatment.

* NC vs. ETEC challenge (10^7^, 10^8^, 10^9^).

## Discussion

F18 enterotoxigenic *E. coli* challenge studies have utilized a range of inoculation dosages, typically from 10^8^ cfu to 10^11^ cfu, which often result in looser stool consistency and diarrhea (8–11, 13). However, substantial variability exists in the incidence, severity, and duration of diarrhea observed across studies (14). Despite this, the relationship between F18 ETEC burden, epithelial attachment, and downstream effects on intestinal function, integrity, and epithelial dynamics, such as proliferation, differentiation, and renewal, remains poorly characterized.

Most F18 ETEC challenge studies last between 13 and 21 days, reflecting the fact that post-weaning diarrhea (PWD) typically emerges within 2 to 3 weeks post-weaning, peaking between 2 and 6 days post-inoculation (6). In this study, a 5-day challenge was conducted in nursery pigs at 10 days post-weaning using varying inoculum doses. Diarrhea was observed in challenged groups between 3 and 5 dpi, particularly in pigs receiving the 10^8^ cfu dose. These findings are consistent with previous reports by Becker et al. (20), Kim et al. (19), and Welch et al. (12). Diarrhea may also play a role in host defense. Although ileum claudin-2 gene expression tended to decrease in ETEC-challenged pigs, Tsai et al. (35) showed that claudin-2-mediated water efflux enhances pathogen clearance.

In K88 ETEC-challenged pigs, Zhu et al. (36) reported concurrent increases in both CFTR mRNA and protein abundance. In the current study, ileal *CFTR* mRNA tended to be higher in pigs challenged with 10^9^ cfu and lower in those that received 10^8^ cfu. However, epithelial CFTR protein was elevated across all ETEC-challenged pigs, particularly in the ileum. The increase in CFTR protein despite variable mRNA expression may be reflective of post-translational regulation driven by F18-associated enterotoxins (LT, STa, and STb) and localization. It is well known that F18 ETEC enterotoxins trigger secretory diarrhea via cyclic AMP-dependent chloride and water secretion through CFTR (1, 37). Once inside enterocytes, these enterotoxins stimulate CFTR channel activation via phosphorylation of regulatory proteins, leading to increased chloride and bicarbonate secretion into the intestinal lumen and subsequent osmotic water efflux that contributes to secretory diarrhea (38–40). Together, these data suggest that CFTR functionality in the presence of F18 ETEC is not always related to transcriptional abundance. Furthermore, consistent with increased CFTR protein localization on the epithelium and bicarbonate and ion secretion, ileal luminal pH tended to be more alkaline in ETEC-challenged pigs compared to NC. A less acidic luminal environment may favor ETEC colonization and contribute to the pathogenesis of post-weaning diarrhea, as previously proposed (41–43).

Confirmation of F18 ETEC-induced PWD requires isolation of hemolytic *E. coli* and confirmation of F18 fimbriae and enterotoxin genes. A strength of this current study was the utilization of qPCR to measure fecal and ileal *F18* and *LT* gene abundance, complemented by ISH to evaluate the localization *F18* transcripts in formalin-fixed intestinal tissues. This combination can be used to enhance diagnostic accuracy (44). In agreement with our hypothesis, fecal *F18* gene transcript was elevated irrespective of treatment in the ETEC-challenged pigs at 5 dpi, confirming successful colonization. Validation of challenge models is often done via fecal culture and PCR for the *FedA* gene. Hansen et al. (45) showed higher *FedA* expression in challenged versus control pigs. Duarte et al. (22) similarly reported increased *FedA* in pigs challenged with 5.2 × 10^9^ cfu. Frydendahl et al. (26) demonstrated bacterial replication above the inoculation dose via blood agar culture. However, these methods lack spatial resolution regarding bacterial attachment. To address this, ISH was employed to test if increasing F18 ETEC challenge dosage would augment localization in intestinal epithelium. In support of this hypothesis, the results revealed that challenged pigs had increased epithelial *F18* transcript localization, indicative of epithelial attachment, in the jejunum, ileum, and colon compared to the NC. Previous studies utilizing F18 ETEC challenge models have primarily focused on the jejunum (18, 22), the ileum (12, 20), or both regions (19, 21). Consistent with the findings of Li et al. (21), the present study found that F18 ETEC epithelial association was greatest in the ileum of challenged pigs at 5 dpi compared to NC pigs. Collectively, these findings demonstrate that ISH provides a valuable diagnostic complement to molecular assays by confirming localized F18 attachment, thereby improving the accuracy and interpretive resolution of PWD diagnosis. Incorporating ISH into experimental and diagnostic workflows can strengthen model validation, improve understanding of infection dynamics, and enhance the development of targeted strategies to reduce the incidence and severity of post-weaning diarrhea.

Consistent with prior research (19, 20, 22), F18 ETEC negatively affects growth performance. Li et al. (21) reported reduced ADG, ADFI, and final body weights at 7 dpi in pigs challenged with 10^9^ cfu, while McLamb et al. (16) found no differences in performance over a 4-day challenge. Similarly, Duarte et al. (18) observed body weight reductions by 13 dpi, but not at 3 dpi. In agreement with these studies, the current 5-day challenge did not significantly affect body weights or performance, although a tendency toward reduced ADG was noted in the 10^8^ cfu treatment. These results suggest that a 3–5-day window may be insufficient to capture the full disease phenotype of F18 ETEC and associated reductions in growth performance.

While F18 ETEC primarily causes secretory diarrhea through enterotoxin release, it also significantly disrupts the intestinal epithelial renewal process, compromising gut barrier function, integrity, and recovery. Infection most often leads to villous atrophy and crypt hyperplasia, as damaged villi are sloughed and crypt cells increase proliferation to compensate (46). Morphologically, ETEC has been shown to reduce villus height and increase crypt depth, resulting in a decreased villus height to crypt depth ratio, indicating immature mucosa and impaired absorption (16, 18). In agreement, jejunum atrophy and decreased villus height-to-crypt depth ratio were reported in the current study after 5 days of F18 ETEC challenge. However, even though the ileum had a greater F18 association, villus height and villus height-to-crypt depth ratios were not different in comparison with the NC treatment.

Following intestinal epithelial damage, crypt cell proliferation can be activated as a regenerative response to restore the integrity and function of the intestinal epithelium. This proliferative activity can be assessed using Ki67 immunostaining, a nuclear marker that identifies actively dividing cells within the intestinal crypts. Although not specific to ETEC, crypt hyperplasia with increased Ki67 staining has been observed under enteric challenge (32). Increased Ki67 staining is generally associated with increased epithelial turnover following enteric inflammation and stress (18). However, ileum Ki67 staining was reduced in pigs challenged with 10^9^ cfu of F18 ETEC, indicating suppression of epithelial renewal. Villous atrophy and reduced proliferation in response to ETEC infection have been previously reported and are associated with enterotoxin-mediated epithelial injury, weaning stress, and inflammatory cytokine signaling (47).

Despite this reduction in proliferative activity, *WNT4* and *β-catenin* mRNA expression, central regulators of crypt stem cell proliferation via the canonical Wnt pathway (24), remained unchanged. This suggests that ETEC-induced suppression of proliferation may be mediated by post-transcriptional regulation or interactions with inflammatory or microbial signals that act downstream of Wnt ligand activation (48). In contrast, components of the Notch signaling pathway, which governs epithelial cell fate and lineage commitment (23), showed signs of dysregulation. Specifically, *Notch* mRNA was elevated in the ileum tissue of F18 ETEC-challenged pigs. Treatments 10^7^ and 10^8^ exhibited approximately 2.0- and 2.1-fold increases in *Notch* expression, respectively, compared to NC, whereas treatment 10^9^ exhibited only a 1.2-fold increase. Conversely, compared to the NC, expression of downstream effector *Hes1* was significantly decreased, particularly in the 10^9^ cfu group. This pattern suggests that treatments 10^7^ and 10^8^ may have activated compensatory signaling to maintain epithelial homeostasis, whereas treatment 10^9^ likely exceeded the tissue’s adaptive capacity, leading to dysregulated Notch signaling and impaired epithelial renewal. This mismatch suggests disruption of canonical Notch activation, which may impair absorptive cell differentiation and barrier maintenance (49).

Markers of secretory lineage commitment and goblet cell function, *ATOH1* and *Muc2*, respectively, remained unchanged, indicating that while the Notch-Hes1 axis is altered, secretory differentiation may be maintained or regulated via alternative pathways. Collectively, these findings are consistent with previous reports that enteric pathogens can interfere with epithelial signaling and cell renewal processes through cytokine modulation, oxidative stress, and tight junction disruption (50–52).

Immature enterocytes derived from intestinal crypts exhibit reduced expression of brush border enzymes (e.g., sucrase, maltase, aminopeptidases) and nutrient transporters (e.g., SGLT1, PEPT1), limiting digestive and absorptive capacity (52, 53). This immaturity, particularly following epithelial injury or accelerated turnover, can contribute to malabsorption and osmotic diarrhea. Additionally, nutrient transporter expression, transcription, and kinetics are known to be influenced by infection and physiological stress. Consistent with observations in other challenge models such as Porcine reproductive and respiratory syndrome virus (31) and heat stress (28), F18 ETEC-challenged pigs exhibited increased ileal *SGLT1* mRNA expression compared to NC pigs. Despite these transcriptional increases, functional nutrient flux assessments using Ussing chamber electrophysiology revealed no corresponding change in active glucose or glutamine transport, suggesting a post-transcriptional disconnect or impaired functional maturation of the enterocytes in response to ETEC-induced epithelial disruption.

Previous studies have shown that ETEC challenge downregulates tight junction proteins, including occludin and ZO-1 (20) and claudin-1 (21, 54), in porcine jejunal and ileal tissues. *In vitro* models using IPEC-J2 cells further demonstrate that ETEC disrupts TER (50), ZO-1 localization, and increased monolayer cell dissociations and disconnections (51), reflecting tight junction perturbation and increased cell shedding. Based on this body of work, we hypothesized that an F18 ETEC challenge would reduce intestinal integrity in nursery pigs. However, no differences were observed in TER or paracellular FD4 flux between F18 ETEC-challenged and NC pigs in the current study. This is consistent with findings from McLamb et al. (16). Together, these data suggest that early-stage F18 ETEC-induced diarrhea may be mediated more by enterotoxin-driven secretory mechanisms than by overt paracellular barrier disruption.

## Conclusion

In agreement with our hypothesis, this study demonstrates that a 5-day F18 ETEC challenge increases diarrhea and bacterial attachment, notably in the ileum. In support of increased diarrhea, epithelial CFTR abundance was increased. However, intestinal barrier function and pig growth performance were not compromised due to early F18 ETEC infection. The ETEC challenge altered ileal markers of stem cell proliferation and Notch pathway signaling, indicating early disruption of epithelial homeostasis, consistent with our hypothesis. These findings support a model in which F18 ETEC attachment triggers diarrhea through an enterotoxin-driven, CFTR-mediated secretory mechanism, rather than through structural damage to the intestinal epithelium. Longer challenge durations may be needed to fully capture the impacts of F18 ETEC on epithelial regeneration, barrier integrity, and growth outcomes.

## Data Availability

The original contributions presented in the study are included in the article/Supplementary material, further inquiries can be directed to the corresponding author.
