# Supplementary material for: Evaluation of F18 enterotoxigenic Escherichia coli intestinal attachment and early disease onset in nursery pigs
Source: Front Vet Sci. 2025 Dec 10;12:1686769. doi: 10.3389/fvets.2025.1686769 (PMC12728359; doi:10.3389/fvets.2025.1686769)
Supplement: Supplementary file 1 [file Table_1.DOCX]

**Additional file 1.** Primer sequences.

| **Gene** | **Sense (5'-3') - forward** | **Antisense (5'-3') - reverse** |
| --- | --- | --- |
| ATOH1 | GAACGGGGTACAGAAGCAAA | TGGACAGCTTCTTGTCGTTG |
| AQP3 | TCGCCAACAACCAGCTTATAG | CTGGTCGAAGAAGCCATTGA |
| CFTR | TTCTGGCGAGCCTTTAGAGAGA | GCTTGAGGGACCAGTGACATTT |
| Cldn2 | AGGCCTCCTGGGCTTCAT | GGAGTAGAAGTCCCGCAGGAT |
| GAPDH | TGGTGAAGGTCGGAGTGAAC | GAAGGGGTCATTGATGGCGA |
| HES1 | AAGGCGGACATTCTGGAAAT | CCTCGTTCATGCACTCACTG |
| IL8 | GCACTTACTCTTGCCAGAACTG | CAAACTGGCTGTTGCCTTCTT |
| MUC2 | CTGTGTGGGGCCTGACAA | AGTGCTTGCAGTCGAACTCA |
| Notch | AAGCCAGCCCTTTACTCCTTTG | CGAACAGCGACGTAACACTGAA |
| SGLT1 | GGCTGTTCCAACATTGCCTA | CAACATGACCGACAGCATCA |
| WNT4 | GACGCCTCTGTGTGTGTTTCTT | TCTGCCTCTGCATCTCCTAACT |
| β-catenin | TCTCCCAGCAACATACGCAG | TACCCGCTCTGACAAACACC |
